# Archaea Symbiont of *T. cruzi* Infection May Explain Heart Failure in Chagas Disease

**DOI:** 10.3389/fcimb.2018.00412

**Published:** 2018-11-21

**Authors:** Maria de Lourdes Higuchi, Joyce T. Kawakami, Renata N. Ikegami, Marcia M. Reis, Jaqueline de Jesus Pereira, Barbara M. Ianni, Paula Buck, Luanda Mara da Silva Oliveira, Marilia H. H. Santos, Ludhmila A. Hajjar, Edimar A. Bocchi

**Affiliations:** Instituto do Coraçao, Hospital das Clinicas HCFMUSP, Faculdade de Medicina, Universidade de São Paulo, São Paulo, Brazil

**Keywords:** heart failure, microvesicles, Chagas disease, exosomes, biomarkers

## Abstract

**Background:** Archaeal genes present in *Trypanosoma cruzi* may represent symbionts that would explain development of heart failure in 30% of Chagas disease patients. Extracellular vesicles in peripheral blood, called exosomes (< 0.1 μm) or microvesicles (>0.1 μm), present in larger numbers in heart failure, were analyzed to determine whether they are derived from archaea in heart failure Chagas disease.

**Methods:** Exosomes and microvesicles in serum supernatant from 3 groups were analyzed: heart failure Chagas disease (*N* = 26), asymptomatic indeterminate form (*N* = 21) and healthy non-chagasic control (*N* = 16). Samples were quantified with transmission electron microscopy, flow cytometer immunolabeled with anti-archaemetzincin-1 antibody (AMZ 1, archaea collagenase) and probe anti-archaeal DNA and zymography to determine AMZ1 (Archaeal metalloproteinase) activity.

**Results:** Indeterminate form patients had higher median numbers of exosomes/case vs. heart failure patients (58.5 vs. 25.5, *P* < 0.001), higher exosome content of AMZ1 antigens (2.0 vs. 0.0; *P* < 0.001), and lower archaeal DNA content (0.2 vs. 1.5, *P* = 0.02). A positive correlation between exosomes and AMZ1 content was seen in indeterminate form (*r* = 0.5, *P* < 0.001), but not in heart failure patients (r = 0.002, *P* = 0.98). Higher free archaeal DNA (63.0 vs. 11.1, *P* < 0.001) in correlation with exosome numbers (*r* = 0.66, *P* = 0.01) was seen in heart failure but not in indeterminate form (*r* = 0.29, *P* = 0.10). Flow cytometer showed higher numbers of AMZ1 microvesicles in indeterminate form (64 vs. 36, *P* = 0.02) and higher archaeal DNA microvesicles in heart failure (8.1 vs. 0.9, *P* < 0.001). Zymography showed strong% collagenase activity in HF group, mild activity in IF compared to non-chagasic healthy group (121 ± 14, 106 ± 13 and 100; *P* < 0.001).

**Conclusions:** Numerous exosomes, possibly removing and degrading abnormal AMZ1 collagenase, are associated with indeterminate form. Archaeal microvesicles and their exosomes, possibly associated with release of archaeal AMZ1 in heart failure, are future candidates of heart failure biomarkers if confirmed in larger series, and the therapeutic focus in the treatment of Chagas disease.

## Introduction

Chagas disease is caused by the protozoan *Trypanosoma cruzi (T. cruzi)*, an endemic infection in Latin America (Strasen et al., 2014; Bocchi et al., 2017), having two distinct phases: acute and chronic. The acute phase is usually asymptomatic or with mild unspecific symptoms (fever, fatigue, malaise, joint pain, and headaches), making it difficult to diagnose (Carvalho et al., 2017). After symptoms of the acute phase resolve, patients can stay asymptomatic for decades; 60–70% of patients have the indeterminate form (IF) for the rest of their lives. However, an intriguing aspect in Chagas disease is why 30% of *T. cruzi*-infected individuals have a worse outcome, with development of heart failure (HF) and sudden death due to a chronic dilated cardiopathy after 10 to 30 years.

Our previous studies with endomyocardial biopsies from chagasic patients showed that HF patients have lymphocytic myocarditis (Pereira Barretto et al., 1986), with lymphocytes injuring non-*T. cruzi* infected myocytes (Higuchi et al., 1987). These patients have myocardium with *T. cruzi* antigens (Higuchi et al., 1993; Bellotti et al., 1996), and DNA (Jones et al., 1993), but in too scarce amount to explain the intensity of inflammatory infiltrate with unbalanced immune response (Cunha-Neto et al., 1995; Gao et al., 2003), suggesting that other factors beyond the parasite may be interfering (Higuchi et al., 1997; Marin-Neto et al., 2007). The presence of other microorganisms could explain this exacerbated inflammation and the lack of response to specific treatment against *T. cruzi* (Rodriques Coura et al., 2002; Morillo et al., 2015). A large number of archaeal-like genes have been described in persistent pathogens conferring metabolic capabilities as adaptation strategies for survival even in hostile host niches (Srinivasan and Morowitz, 2006). In reviewing endomyocardial biopsies studied using transmission electron microscopy (TEM), we identified archaeal DNA in microvesicles (Higuchi et al., 2009). *T. cruzi* may carry archaeal genes because the parasite has an archaeal-like M32 metallocarboxypeptidase and proteasomes consistent with a common ancestor archaeal-eubacteria (Gille et al., 2003; Niemirowicz et al., 2007). This suggests that archaea might have a role in the development of HF, with a strong pathogenic impact. Archaea are the oldest microorganisms described in nature, frequenty considered non-pathogenic, but is capable of increasing inflammation, and development of dieases (van de Pol et al., 2017). Archaea are known to entrap proteins by generating an increased immune response of CD8^+^ T lymphocytes, without activation of CD4^+^ T (Krishnan et al., 2000), a characteristic also present in chagasic myocarditis (Higuchi et al., 1997).

Although originally considered artifacts or a sign of cell death, in recent years interest has increased in the study of extracellular vesicles (EVs) as mediators of long-range cellular communication because of their presence in most body fluids. According to their size, EVs are classified in microvesicles (0.1 to 1 μm) or exosomes (< 0.1 μm).

Microvesicles and exosomes may be protective, promoting cellular survival by removing dangerous or redundant intracellular compounds (van der Pol et al., 2010). Other studies show that when released by most of the cells they may contribute to the distribution of cancers and infectious diseases through delivering the pathogenic agents to the uninfected cells (Bakhshandeh et al., 2017) and may also have a role in the development of heart failure (Pfeifer et al., 2015).

In the present work, we investigated whether serum microvesicles and exosomes containing archaeal DNA or archaeal collagenase are related to HF in Chagas disease patients.

## Materials and methods

The study was carried out using serum samples from Chagas disease patients collected from 2008 until 2016 and patients without cardiac disease (negative control group) at our institute. For the HF group, the inclusion criterion was patients with heart failure according to Framingham criteria, i.e., < 35% systolic left ventricle ejection fraction. The inclusion criterion for the IF group was asymptomatic patients based on the presence of two positive serologic tests for Chagas disease in combination with a normal electrocardiogram, chest radiograph with no evidence of cardiac enlargement, neither esophagus, or colon enlargement by contrasted exams and ejection fraction >60%. Transthoracic echocardiogram was performed by using the commercially available system (Envisor Philips, Philips Healthcare, Bothell, USA). All the measurements were performed and reported according to the recommendations of the American Society of Echocardiography (Lang et al., 2015). We assessed the following echocardiographic parameters: left atrium diameter, interventricular septum diameter, posterior wall thickness, LVEF, LV end-diastolic diameter (LVDD), LV end-systolic diameter (LVSD; Lang et al., 2015). Experienced board certified echocardiographers in ICESP performed all echocardiograms.

Approximately 10 ml of blood was collected in a dry tube from 25 HF, 21 IF and 16 CTL patients. The tubes remained for 1 h at 37°C, and then were centrifuged at x250 g for 5 min. Sera were collected in microtubes and stored at −20°C until phase separation.

### Separation and isolation of microvesicles and exosomes

This isolation method was performed according to the procedure used to separate mitochondria (Bustamante et al., 2005) modified by us, which made possible the high recovery of microvesicles and exosomes from sera, detected with TEM.

Sera were diluted (1:5) in medium containing 200 mm D-Mannitol (Fresenius Kabi Brazil, Aquiraz, CE, BR), 70 mm sucrose (EMS Electron Microscopy Sciences–Hatfield, PA, USA), 2 mm Hepes (Fair Lawn, NJ, USA) and 0.5 g/L BSA pH 7.2 (Sigma, St. Louis, MO, USA), and incubated for 1 h at room temperature (RT). The samples were then centrifuged for 12 min at x9500 g and supernatants were collected for processing for Transmission Electron Microscopy (TEM).

### Fast inclusion method for transmission electron microscopy

Inclusion was done following a fast procedure (Duarte et al., 1992), modified by us as described below:

After the separation phase, to detect exosomes present in the supernatant, 400 μl of the supernatant fraction was fixed by adding 1 ml of 3% glutaraldehyde at 4°C for 3 h, postfixed by adding reduced osmium solution (700 μl of 1% osmium tetroxide solution + 700 μl of potassium ferrocyanide 3%) at 4°C for 30 min and centrifuged x13,000 g for 5 min at 12°C. This procedure made a pellet formation possible, as the exosomes and lipidic microvesicles present in the supernatant became heavier after fixation. Then, the supernatant pellets were washed in a solution containing 0.9% NaCl + 360mOs of saccharose and incubated in 0.5% uranyl acetate for 3 h at 4°C. Pellets were dehydrated for 10 min in 70% ethanol, 5 min in 0.1N acidified 2,2-dimethoxypropane with HCl, followed by remaining for 2 min in acetone in 4% copper sulfate.

The infiltration was done with a mixture containing epon EMbed 812 resin with araldite resin 502 (1:1) and oven polymerization 100°C for 1 h. The blocks were cut in ultramicrotome with thickness between 60 and 70 nm and placed in 200 mesh copper and nickel-plated grids of Parlodium (all reagents and grids were from Electron Microscopy Sciences, Hatfield, PA, USA).

### Microvesicles and exosomes characterization at TEM

Copper grids were contrasted with 0.08 M lead citrate solution for 5 min according to technique described by Reynolds (Reynolds, 1963) and photographed in 50,000 × magnification in TEM. Four photos of each case were analyzed in areas of higher exosomes/ microvesicles concentration, evaluating number of exosomes (0.02 to 0.1 μm) and microvesicles (0.1 to 1 μm).

### Flow cytometer

Quantification of microvesicles (<0.1 μm) in sera was also performed by flow cytometer adapted from previously described protocols (Bode and Hickerson, 2000; Couper et al., 2010) by calibration with defined-size fluorescence microbeads (0.79 to 1.34 μm Spherotech Inc., Libertyville, IL, USA).

Identification of archaea DNA in microvesicles was performed using the hybridization technique, generic probe ARCH 915. Each 25 μl serum sample was incubated with a probe (40 ng/μl) in an oven at 45°C for 20 h. Then it was incubated with PE-Texas Red Streptavidin (BD Pharmigen™, USA) for 30 min RT, protected from light. In addition, mouse IgG antibodies labeled with FITC and PE (isotype control) were used as controls. Identification of microvesicles with metalloproteinase from Archaea (AMZ1) was performed by incubation with anti-AMZ1 primary antibody (4 ng/μl, Novus Biologicals, Littleton, CO, USA) for 1 h, RT, and then incubated with secondary antibody (Donkey anti-Rabbit IgG (H+L) Secondary Antibody, Alexa Fluor 555) for 30 min RT, protected from light.

Samples were analyzed using a flow cytometer (FACSCalibur-Becton-Dickinson, California, USA), where about 10,000 events were obtained in each sample, with at least 5.000 events within the specific region for microvesicles. The analysis was done using FlowJo software (Tree Star).

### Zymography

This procedure was used to determine the levels of AMZ1 (metalloproteinase from Archaea) in samples of supernatant sera from chagasic patients and control group. Approximately 80 ug of protein from each sample were subjected to electrophoresis on the polyacrylamide gel. Samples' concentration was determined by Bradford method (Bradford, 1976) and calculated according to the equation of the line obtained from a concentration curve. Samples were incubated with Novex™ Tris-Glycine SDS Sample Buffer 2x Buffer (Thermofisher; without the presence of reducing agent or heating in order to maintain the function of the enzymes present). All samples were loaded onto 12% polyacrylamide gel along with 2 mg/ml casein (C-0376 Sigma) as it is Archaea-specific protease substrate. After electrophoresis, the gel was washed with 1X renaturing buffer containing non-ionic detergent, 2.5% Triton X-100 (Novex zymogram renaturing buffer–Invitrogen) for 30 min at room temperature under gentle agitation. Subsequently, the gel was incubated in Tris buffer for 30 min at room temperature. This incubation was repeated for 48 h at 37°C, allowing digestion of the substrate by the protease. The gel was washed with deionized water and stained for 1 h with Comassie Blue R-350, placed on a 300-dpi, or higher under-resolution plastic scavenger. The band intensities were determined by densitometry using the UVITEC Aliance Q4- 365 Advanced Imaging System software. The digested clear bands were normalized to protein β-actin values, each gel having HF, and IF samples, and control samples which values were considered as 100%, and were the base to obtain the quantification of other two groups.

### Electron microscopy immunolabeling technique

To remove the resin and expose the masked antigenic sites, grids were incubated in 0.5 M sodium metaperiodate solution (Sigma, St. Louis, MO, USA) for 1 min RT, washed 4 times in distilled water and incubated in CAS-block (Life Technologies, Frederick, MD, USA) for 30 min RT.

They were then incubated with anti-AMZ-1 primary antibody (4 ng/μl, Novus Biologicals, Littleton, CO, USA) for 16 h at 4°C, washed in phosphate buffered saline (PBS), incubated for 1 h RT with diluted secondary antibody labeled with 10 nm gold particles (EMS—Electron Microscopy Sciences, Hatfield, PA, USA).

The grids were contrasted with 0.08 M lead citrate solution for 5 min according to the technique described by Reynolds (Reynolds, 1963), for later observation on TEM.

### *In situ* hybridization electron microscopy

In some of the cases (9 HF−07 males and 13 IF−04 males) supernatant samples were studied by *in situ* hybridization at TEM, using 40 ng/μl archaea DNA biotinylated generic probe (ARCH915, GTGCTCCCCCGCCAATTCCT), incubated at 45°C overnight. They were then incubated for 1 h RT with diluted secondary antibody labeled with 10 nm streptavidin gold particles (Sigma, USA). The numbers of exosomes and archaea DNA dots were counted in 4 photos representing the richest places of each case in x50K magnification.

### TEM analysis

Analysis was made using TEM photographs, quantifying exosomes, AMZ-1 antigens, and archaeal DNA.

The mean numbers of exosomes, AMZ1, and archaeal DNA-positive dots inside and outside microvesicles were obtained by counting 4 photos representing the richest places of each case in x50K magnification.

### Statistical analysis

Comparison between HF, IF, and Control group was performed by ANOVA and Multiple Comparisons using Dunn's Method. Spearman's Correlation Analysis was used to find correlation between AMZ1 antigens and archaeal DNA in Flow Cytometer. Results were expressed as median values.

## Results

### Studied populations

Chagasic group was composed by patients with mean age of 54 ± 12 years, median of 55 years, varying from 30 to 85 years. Heart failure group presented 49± 10 years, 20 males (mean age 49± 9 years) and 5 females (mean age 51 ± 14). IF group presented mean age of 60 ± 12 years, 9 males (mean age 65± 12) and 12 females (mean age 57± 11).

Non-chagasic healthy control group was composed by 16 individuals, mean age 56 ± 11 years, varying from 35 to 70 years, median of 60 years. There were 11 males (mean age 61 ± 9 years) and 5 females (mean age 45 ± 7 years).

### Transmission electron microscopy

Median numbers of exosomes (vesicles < 100 nm) in IF, HF, and Control groups were, respectively 275, 74, and 84, significantly increased in IF compared to other two groups using Multiple comparison test (*P* < 0.001). There was difference between IF vs. control and IF vs. HF, but no difference between HF with control group (Table 1).

**Table 1:** Median numbers of microvesicles in sera by flow cytometer with AMZ1 antigens and archaeal DNA.

	**Flow Cytometer**		**TEM**		**Zymography**
**Groups**	**Microvesicles AMZ**	**Microvesicles Archeal DNA**	**Exosomes**	**Microvesicles**	**AMZ activity**
HF (*n* = 26)	36	8	74	1	117
IF (*n* = 21)	64	0.9	275	0	103
Control (*n* = 16)	44	5	84	0	100
P^*^	0.01	<0.001	<0.001	<0.001	<0.001

Median numbers of exosomes and microvesicles by TEM and percentages of microvesicles with archaeal collagenase activity (AMZ) by Zymography in HF, IF, and Control groups.

* *ANOVA*.

Microvesicles (vesicles >100 nm) mean numbers in IF, HF, and control groups were, respectively 1.6 ± 3.7, 3 ± 5 and 0.2 ± 0.7, median numbers 0, 1 and 0. HF had significantly increased numbers than IF and control (*P* < 0.001). There was difference between HF vs. control and IF vs. HF, but no difference between FI with control group (Table 1).

**Figure 1:**
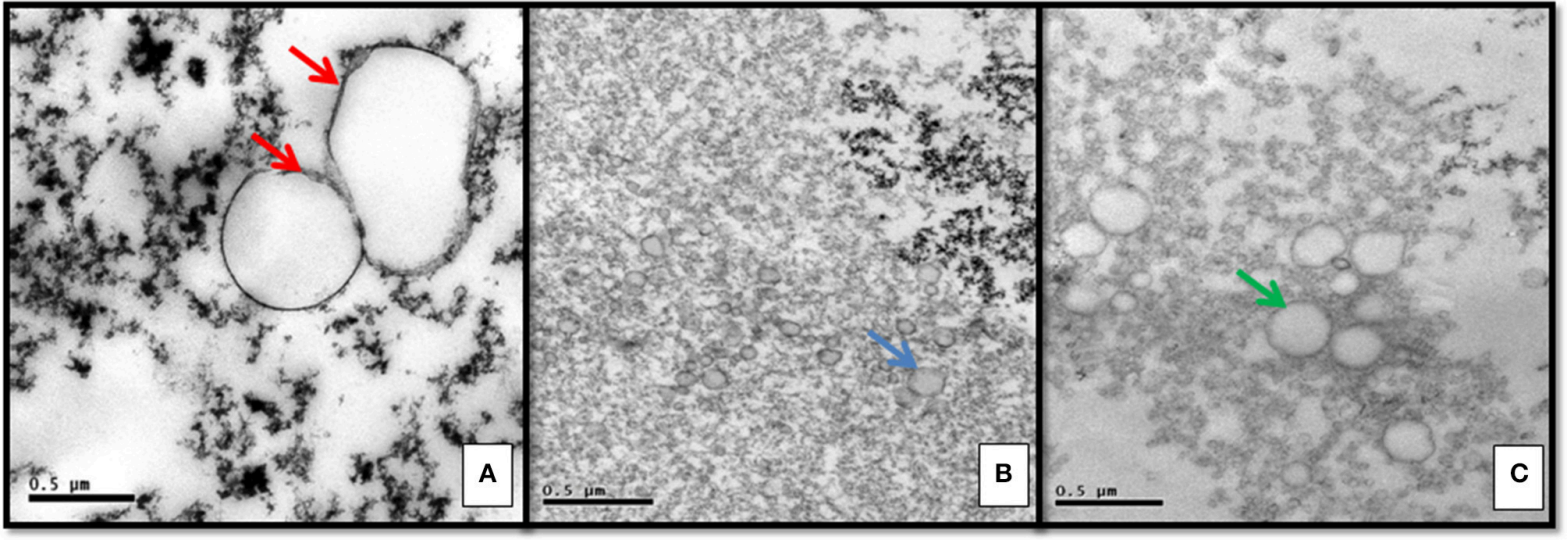
Ultrastructural morphology of vesicles in serum supernatant: **(A)** Heart failure chagasic form exhibiting pathogenic archaea with double membrane surrounding clear vesicles (red arrows). **(B)** Indeterminate form with electron dense protective microvesicles (blue arrow). **(C)** Negative control group exhibiting lipidic microvesicles, surrounded by monolayer membrane (green arrow).

Ultrastructural morphology of microvesicles present in HF was different from those present in IF (Figure 1). HF had larger and clear microvesicles, surrounded by two envoltory membranes, compatible with archaea. IF presented electron dense microvesicles, which were suggestive of human protective microvesicles (Figure 1).

### Flow cytometer

Quantification of microvesicles in the sera by flow cytometer [calibrated with 0.79 to 1.34 μm microbeans) showed significantly higher median percentages of microvesicles with AMZ1 antigens in IF than in HF and control groups (64, 36 and 43) *P* = 0.01].

There were lower numbers of microvesicles with archaeal DNA in IF than in HF group and control group (0.9, 8 and 5 with *P* < 0.001).

In both, AMZ1 and archaeal DNA quantification, there was statistically significant difference between IF vs. control and IF vs. HF, but no difference between HF with control group (Table 1).

A significant negative correlation between AMZ1 antigens vs. archaeal DNA was seen in HF (*r* = −0.5, *P* = 0.009) and a positive correlation with control group (*r* = 0.55, *P* = 0.02) but not in IF group (*r* = −0.005, *P* = 0.98).

### Zymography

The result of zymography using casein as Archaea-specific protease substrate, using the control group as 100% of collagenase activity showed significantly increased activity in HF compared to IF and control groups (121 ± 14, 106 ± 13 and 100), median, respectively 117, 103 and 100, *P* < 0.001.

### Immunoelectron microscopy

After the immunolabeling technique, ultra structural analysis of serum supernatant showed that asymptomatic IF patients had higher median numbers of exosomes (58.5 vs. 25.5, *P* < 0.001), than HF group. Exosomes in IF group had higher amounts of AMZ1 antigens than exosomes in HF group (2.0 vs. 0; *P* < 0.001). A significant positive correlation between numbers of exosomes and their content of AMZ1 antigens was seen in IF (*r* = 0.5, *P* < 0.001), but not in HF group (*r* = 0.002, *P* = 0.98). The amount of AMZ1 antigens outside exosomes did not differ between IF (6.0) and HF groups (4.0), *P* = 0.72.

### *In situ* hybridization electron microscopy

The exosomes in IF contained higher amounts of archaeal DNA dots (1.5 vs. 0.2, *P* = 0.02). HF had greater mean number of extracellular archaeal DNA dots than IF group (63.0 vs. 11.1, *P* < 0.001) and had a positive correlation with the number of exosomes (*r* = 0.66, *P* = 0.01), but not in IF group (*r* = 0.29, *P* = 0.10).

## Discussion

The microbial community is considered as having decisive in determining health and disease susceptibility, usually in the gut (Hardison et al., 2017; Manrique et al., 2017).

Infectious agents carrying symbionts may also cause different disease susceptibity. Archaeal genes present in *Trypanosoma cruzi* may represent archaeal symbionts that would explain development of heart failure in 30% of chagasic patients. A characteristic of archaea is that they increase inflammation because of the presence of T CD8+ lymphocyte antigens without activation of CD4+ T cells, as occurs in chagasic myocarditis (Reis et al., 2000). In chronic chagasic myocarditis, microvesicles surrounded by a double membrane containing archaeal DNA were seen in myocardiocytes and at the extracellular matrix surrounded by macrophages and lymphocytes, suggesting that they would be the cause of myocarditis in the absence of *T.cruzi* (Higuchi et al., 2009).

Exosomes are vesicles homogeneous in shape and size, may reduce cardiomyocyte apoptosis and improve myocardial ejection fraction (Gray et al., 2015). In response to stimuli, exosomes act as intercellular signaling organelles involved in several physiological and pathological processes (Corrado et al., 2013), and increased number of exosomes may be related to good outcomes in IF chagasic patients. The possibility that *T. cruzi* may carry archaeal genes is shown by the presence of an archaeal-like M32 metallocarboxypeptidase and proteasomes consistent with a common ancestor archaeal-eubacterial in *T. cruzi* (Gille et al., 2003; Niemirowicz et al., 2007). M32 metallocarboxypeptidase is important for degradation of myocardial cell proteins during intracellular trypanosome growth; archaeal proteasomes are capable of degrading aggregation-prone proteins, reducing their cellular toxicities in mammalian cells (Yamada et al., 2006), and decreasing myocardial inflammation. Proteasomes are organelles that rid the cells of abnormal proteins. Trypanosomes carry proteasomes from archaea probably by an evolutionary endosymbiotic mechanism (Srinivasan and Morowitz, 2006). The essential role of proteasomes in the activity for degrading the cytoplasmic proteins has been previously demonstrated (González et al., 1996; De Diego et al., 2001).

Microvesicles are membrane-coated vesicles, participate in the production of free radicals (Leroyer et al., 2007; Christersson et al., 2017), in intracellular communication, homeostasis, and immunity, acting in different pathological processes including inflammatory response, which would make them possible biomarkers of acute events (Barry et al., 1998; Huber et al., 2002; Brodsky et al., 2004; Ridger et al., 2017). Microvesicles are abundantly released in patients with cardiovascular diseases, tumors, and neurological diseases; microvesicles in the serum might be diagnostic and/or prognostic (Colombo et al., 2012; Xiong et al., 2012).

In the present work we found that serum microvesicles may also be derived from microbes, such as archaea. We compared the numbers of exosomes and microvesicles in the serum of HF and IF chagasic patients, and healthy non-chagasic patients. We found increased numbers of exosomes, containing AMZ1 in IF compared to HF chagasic and healthy control non-chagasic individuals. Archaeal DNA microvesicles were seen in increased numbers in HF and healthy group compared to IF. We could also differentiate the healthy control vs. chagasic patients by quantification of active collagenase in the serum, that was strongly increased in HF group and mild increased in IF compared to healthy control group. Most of normal individuals may carry archaea, but only some are associated with disease (van de Pol et al., 2017). This may be explained by the presence of pathogenic archaea, releasing active collagenase.

A large number of archaeal-like genes were described in persistent pathogens conferring metabolic capabilities as adaptation strategies for survival even in hostile host niches (Díaz-Perales et al., 2005; Srinivasan and Morowitz, 2006) evaluated the possibility that human tissue could produce proteases previously described in evolutionarily distant organisms but had not been described in mammals. They identified two new human metalloproteases called archaemetzincin (AMZ1 and AMZ2) that are widely distributed in Archaea and vertebrates, which we found increased in chagasic patients. Archaea may also release exosomes; in this study we demonstrated that exosomes containing archaeal DNA were increased in the HF compared to IF patients. On the other hand, larger numbers of exosomes in IF, frequently containing AMZ1 collagenase and a large quantity of them in the extracellular space at the supernatant, suggests a protective role for human exosomes, degrading AMZ1 archaeal collagenase. However, exosomes released from pathogenic archaea, are superior adjuvants that induce long-term CD8+ cytotoxic T cell response to entrapped soluble protein in the absence of CD4+ T cell help (Krishnan et al., 2000), releasing active collagenase.

Immunoadsorption treatment to remove cardio toxic autoantibodies in patients with chronic inflammatory dilated cardiomyopathy improved endothelial and myocardial function in association with a significant drop in circulating microvesicles (Bulut et al., 2011). Also, several articles in the literature report that exosomes and microvesicles may contribute to delivering pathogenic agents to no infected cells and may have a role in the development of heart failure.

We propose that these human exosomes are protective, removing and destroying the archaeal collagenase, functioning as proteasomes, and preventing heart dilatation. Fewer numbers of protective exosomes in HF group are probably not capable of degrading AMZ1. Re-enforcing this idea, flow cytometer analysis demonstrated larger numbers of microvesicles positive for archaeal DNA in HF sera, and zymography demonstrated increased archaeal collagenase activity. Larger numbers of human exosomes containing AMZ1 antigens in IF are possibly being removed from circulation to be degraded.

## Conclusions

This study suggests that different outcomes of Chagas disease are not related to *Trypanosoma cruzi* but to the presence of its symbiont, the archaea, and archaea nanovesicles like exosomes. HF was associated with increased numbers of microvesicles and exosomes containing archaeal DNA, apparently releasing free archaeal DNA and AMZ1 antigens, causing increased collagenase activity. HF also has lack of protective exosomes, while IF serum supernatant have increased numbers of them. IF exosomes do not contain archaeal DNA and may have a protective role in removing abnormal proteins (AMZ collagenase), preventing development of HF.

The therapeutic treatment for Chagas disease should therefore include the elimination of pathogenic archaeal and, consequently, archaeal exosomes.

## Study limitations

The results are based on a small cohort of chagasic patients. Further studies involving a larger number of chagasic patients and healthy individuals are necessary for a better understanding of the meaning of exosomes and microvesicles, to confirm whether archaeal microvesicles could be biomarkers of a worse outcome in Chagas disease or a new target for heart failure therapy.

## Ethics statement

The study received approval from the Ethics Committee for Analysis of Research Projects of the Clinical Hospital of the Medical School of the University of São Paulo under number 0268/10, and all patients signed an informed consent form.

## Author contributions

MH, LH, and EB conceived and designed the experiments. JK, RI, and JP performed the experiments. JK and MR analyzed the data. BI, PB, and LO contributed reagents, materials, and analysis tools. MS, MR, and MH prepared the manuscript. MH, LH, EB, and RI revised the manuscript.

### Conflict of interest statement

The authors declare that the research was conducted in the absence of any commercial or financial relationships that could be construed as a potential conflict of interest.
